# Awareness: An empirical model

**DOI:** 10.3389/fpsyg.2022.933183

**Published:** 2022-12-09

**Authors:** Federico Bizzarri, Alessandro Giuliani, Chiara Mocenni

**Affiliations:** ^1^Department of Information Engineering and Mathematics, University of Siena, Siena, Italy; ^2^Environment and Health Department, Istituto Superiore di Sanità, Rome, Italy

**Keywords:** tacit knowledge, cognition, decision making, uncertainty, Markov models, machine intelligence, optimization, intuition

## Abstract

In this work, we face the time-honored problem of the contraposition/integration of analytical and intuitive knowledge, and the impact of such interconnection on the onset of awareness resulting from human decision-making processes. Borrowing the definitions of concepts like intuition, tacit knowledge, uncertainty, metacognition, and emotions from the philosophical, psychological, decision theory, and economic points of view, we propose a skeletonized mathematical model grounded on Markov Decision Processes of these multifaceted concepts. Behavioral patterns that emerged from the solutions of the model enabled us to understand some relevant properties of the interaction between explicit (mainly analytical) and implicit (mainly holistic) knowledge. The impact of the roles played by the same factors for both styles of reasoning and different stages of the decision process has been evaluated. We have found that awareness emerges as a dynamic process allowing the decision-maker to switch from habitual to optimal behavior, resulting from a feedback mechanism of self-observation. Furthermore, emotions are embedded in the model as inner factors, possibly fostering the onset of awareness.

## Introduction

Aside from the classical analytical perspective, this work mainly focuses on the weight of the impact and relevance of other facets that belong to the decision-making processes, such as tacit knowledge, intuition, emotions, awareness, and self-awareness. These factors, once considered largely irrelevant (if not a nuisance to be eliminated) in the decision-making process, are now being taken into account more seriously in cognitive studies, and their multi-faceted effects are intensely analyzed. Even though all of them have been thoroughly studied and described from a theoretical point of view in different fields of investigation, a formalization from a modeling point of view is still missing. This is the gap the present work aspires to begin filling by proposing a possible modeling formulation that is both broad enough to consider all these different aspects and sufficiently simple to be clearly understandable, and thus, interpretable according to the different perspectives of the practitioner. Although limited and imperfect by nature, also due to the difficulties in modeling complex phenomena—as human decisions are—this study could contribute to introducing new aspects which can expand research in the field of decision-making.

This research has a multidisciplinary intent, involving different disciplines ranging from behavioral and cognitive sciences, economy, mathematics, philosophy, and psychology, and attempts to incorporate all the different perspectives involved in the process. We tried to be as faithful as possible to the different concepts exposed according to specialist literature while maintaining a constant interest and focus on the modeling perspective. Indeed, this work does not intend to give unique and definitive modeling recipes; on the contrary is aimed at fostering general interest in the explicit inclusion of crucial aspects of decision-making into quantitative models of awareness. Specifically, our aim is to propose a mathematical model incorporating all processes fostering the emergence of awareness. On one hand by identifying the main characteristics behind decision-making (including analytic and intuitive factors), their relationship with emotions and other drivers, and on the other hand by highlighting novel logical and philosophical aspects such as the importance of tacit knowledge, the correlation between optimal decisions and uncertainty, and the awareness dynamics. The mathematical formulation of the model is grounded on Decision Markov Processes, which embed most characteristics of human decision-making, indeed they integrate control actions, uncertainties, and temporal dynamics. Specifically, at each time step the decision-maker's specific choice provokes a change in the system's state according to the control actions he/she chooses. Additionally, this change is affected by external noisy stimuli. The evolutionary dynamics of such a system can be evaluated for very long-time processes (up to the limit of infinite time). For finite time-horizon processes, such as the ones reasonably considered in this paper, the trajectory of the system depends on the reward functions, the transition probabilities, and, at the final time, the terminal reward value. In any case, the convergence of the trajectory will be evaluated on average due to the stochastic nature of the process.

The formulation of mathematical models enables us to perform extensive simulations to understand the multifaceted nature of this process.

The paper is organized as follows. Section Decision-making processes: an overview exposes general concepts related to decision-making processes and awareness, as the individual styles and approaches, the role of emotions, tacit knowledge, and the relationship between decisions and information. Furthermore, we will offer a panoramic view on how scientific literature deals with the concept of awareness and its impact on decisions. Section Developing a mathematical model of awareness reports the mathematical formulation of the proposed model. It is first simply and generically described, and later possible developments and extensions are introduced. Section Numerical results presents some numerical results of the simulations carried out by applying the proposed model and their discussion.

## Decision-making processes: An overview

### Rationality, intuition, tacit knowledge, and emotions

#### Rationality and intuition

Until very recently, scholars and practitioners agreed that effective decision-making occurs only under the most rational conditions. Since Descartes (2008) cognition has had a stronghold as being the only legitimized contributor to reasoning in that decisions must come exclusively from rational, cognitive, and logical processes, while emotions, intuition, and other subjective aspects are not considered as having a significant role in the process. This conventional teaching has been that “the more objective and rigorous our thinking processes are, the better our decisions will be.” A totally different perspective (much more similar to contemporaneous views) on cognition was outlined in the same years by another French scientist and philosopher, Blaise Pascal (2012). It is worth reporting a small extract of his considerations on cognition processes, which are at the very basis of our proposal (emphasis added).

“*THE DIFFERENCE between the mathematical and the intuitive mind.—In the one, the principles are palpable, but* removed from ordinary use; *so that for want of habit it is difficult to turn one's mind in that direction: but if one turns it thither ever so little, one sees the principles fully, and one must have a quite inaccurate mind who reasons wrongly from principles so plain that it is almost impossible they should escape notice […] But in the intuitive mind*, the principles are found in common use *and are before the eyes of everybody. One has only to look, and no effort is necessary; it is only a question of* good eyesight, *but it must be good, for the principles are so subtle and so numerous, that it is almost impossible but that some escape notice […] These principles are so fine and so numerous that a very delicate and very clear sense is needed to perceive them and to judge rightly and justly when they are perceived*, without for the most part being able to demonstrate them *in order as in mathematics;* because the principles are not known to us in the same way, and because it would be an endless matter to undertake it. We must see the matter at once, at one glance, *and not by a process of reasoning, at least to a certain degree […]”*.

Based on the above statements, it is clear that Pascal considered “intuition” (“*esprit de finesse”* in French, as opposed to the “*esprit de géométrie*”) a proper form of knowledge and not “irrational” and “purely emotive” nuisances to the proper way of reasoning. In another part of his essay, he clearly states that a real scientist (philosopher) must adopt both attitudes to have a fruitful approach to science. In real life we have never had problems accepting Pascal's view, and it is shared common sense to appreciate both the “holistic-intuitive” and “logical-analytic” capacity of decision-makers (whether they are managers, scientists, physicians, etc.). However, shifting from real-life to academic formalism, things abruptly change. The Cartesian way of thinking, much more primitive with respect to Pascal, has been reinforced in more recent times with the ascendancy of empiricism and positivism (Tayor, 2004).

Given the gaps in the rational theories, an alternative perspective proposed by theorists calls for a richer conception of the decision-maker accounting for the assumption that decisions are also driven by emotion, intuition, imagination, experience, and memories, and thus implicitly reviving Pascal's statements. There is now a consistent body of research delving into the nature of decision-making, particularly into the role of cognition, intuition, and emotion in human decisions (Soosalu et al., 2019). Notably, intuition and cognition deal with two different ways to process information, which we call *intuitive* and *analytical* (Adinolfi and Loia, 2021). Although dual-process theories come in several forms, they reflect the generic fundamental distinction between the two processes. The first is related to intuition, which is often considered relatively undemanding in terms of cognitive resources, and is associative, tacit, intuitive, and holistic; hence, at odds with Pascal's position as he considers intuitive and analytical knowledge to be of equal importance. On the contrary, the second involves conscious, analytical, deliberate, cognitive, logical, linear, and reason-oriented thinking, making certain demands on “cognitive” resources (Hodgkinson et al., 2008; Kahneman, 2017). In our opinion, this consideration holds true if (and only if) we consider such resources in terms of computational cost and time, but not in terms of depth and subtlety. It is no accident that the English translation of “esprit de finesse” as “intuition” conveys a somewhat different meaning. The etymological roots of the term *intuition* stem from the Latin word *in-tuir*, which can be translated as “looking, regarding or knowing from within”. Intuition encompasses a complex set of interrelated cognitive, affective, and somatic processes in which there is no apparent intrusion of deliberate, rational thought. Moreover, the outcome of this process (an intuition) occurs almost instantaneously and can be difficult to articulate. The outcomes of intuition are perceived as a holistic “hunch”, a sense of calling or overpowering certainty, and an awareness of knowledge that is on the threshold of conscious perception (Bechara and Damasio, 2005). In their comprehensive review of literature on intuition within the field of management, Dane and Pratt defined intuition as “*affectively charged judgment that arises through rapid, non-conscious, and holistic associations*” (Dane, 2007; Adinolfi and Loia, 2021). We can say that Blaise Pascal's cognition theory has obtained its deserved consideration after nearly 350 years.

#### Tacit knowledge

**Figure 1:**
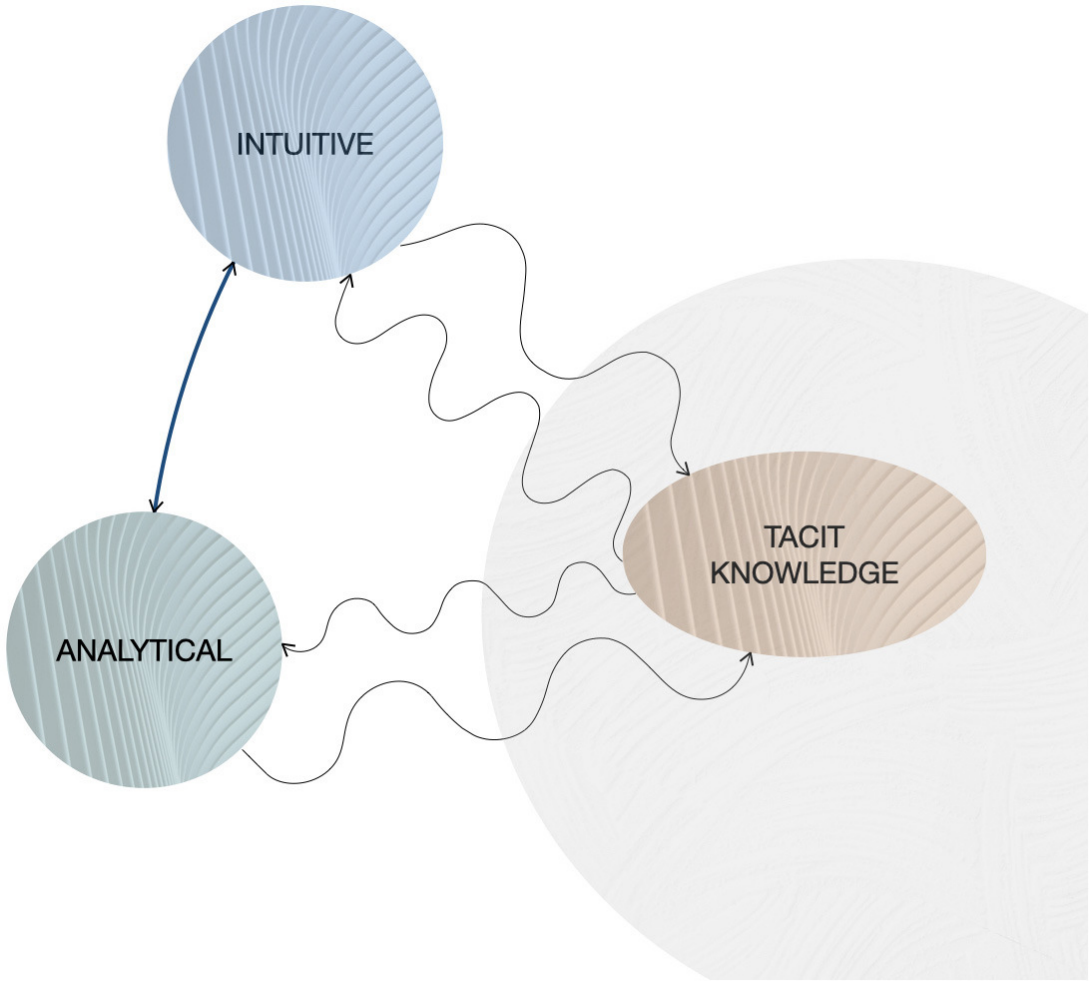
A schematic representation of Knowledge. Tacit knowledge is involved in both intuitive and analytical reasoning, yet it is more heavily used in the former while in the latter it is only slightly adopted. An example of the application of tacit knowledge in analytical reasoning could come from the field of data analysis, where we can recognize the action of tacit knowledge in the choice of the model to use to analyze collected data. In both cases it is a *fast* process: tacit knowledge is immediately available and instantaneously applied by the individual. On the other hand, both intuitive and analytical approaches, thanks to the experience they bring to the individual, contribute to the *slow* process of knowledge sedimentation that creates an individual's unique implicit knowledge. These aspects are represented in the image by links: the *fast* (*slow*) characteristic is figuratively represented by waves with a high (low) frequency. Their amplitude represents the weight of the influence between tacit knowledge and analytical/intuitive reasoning and, on the contrary, the influence of analytical/intuitive approaches on tacit knowledge. Moreover, the characteristic of tacit knowledge that can be neither explicitly declared nor open to introspection is represented by the haziness of its area. The last arrow, between analytical and intuitive, stands for the continuously evolving relationship between these two individual modalities, which has a speed of change entirely depending on individual characteristics.

The roles of intuition and tacit knowledge were formally incorporated into the theory (Brockmann and Anthony, 1998) as factors that lead to better decisions compared to those relying solely on the rational or analytical approach. This is particularly evident in those areas involving creativity such as innovating, visioning, and planning. The concept of *tacit knowledge* (sometimes also called *implicit knowledge*) is mainly attributed to Michael Polanyi, who introduced it for the first time in 1958 in his work Personal Knowledge (Polanyi, 2009). This term indicates a kind of knowledge that is opposed to formal and codified explicit knowledge; it is difficult to express or extract and thus even more difficult to transfer to others through writing or speech (Polanyi, 2009). It represents a content that is neither part of one's normal consciousness nor open to introspection. When applied, tacit knowledge is helpful but not externally expressed or declared; rarely do we recognize when we are using tacit knowledge. According to Polanyi, people cannot describe their use of tacit knowledge; “*we simply know more than we can tell”* is his typical expression to explain this concept. Implicit learning and implicit knowledge contribute to the knowledge structures upon which individuals draw when making intuitive judgments (see the visual representation reported in Figure 1). However, although they may underpin the non-conscious cognitive, affective, and somatic processes that lead to an intuitive judgment, they are not equivalent to intuition (Reber, 1993; Hodgkinson et al., 2008). In our opinion, tacit knowledge is the closest relative to Pascal's “*esprit de finesse*” which, in its original definition, is devoid of any “emotional” aspect.

#### Emotions

As Damasio points out, an important aspect of the purely rational position is that to obtain the best results we must keep emotions out (Damasio, 1994). For a long time, emotion has been largely banished from the predominant philosophies and theories regarding decision, reason, and management. However, emotions have a considerable impact on an individual's decisions and must be carefully considered, particularly the *immediate emotions*. Immediate emotions are those experienced at the moment of decision, in contrast to the ones expected to be experienced in the future, like regret and disappointment (Loewenstein, 2000).

We most certainly always mix emotion and reasoning, analytic attitude and intuition, this mixture being something neuroscientists consider essential (Damasio, 2012). Rational decision-making skills are required to clearly and logically process available information, and thus allow for accurate perception and interpretation of events. In addition to this type of knowledge it is essential to consider that people make decisions based on tacit knowledge grounded in experience, and may use intuitive decision strategies almost exclusively, particularly under high-stress conditions (Sayegh et al., 2004). All these aspects are, to some extent, included in the model proposed in this article, which integrates the analytical, logical, and cognitive abilities of the decision-maker but also subjective aspects like intuition, tacit knowledge, and emotions. These components are always present in any decision-making process and must be collectively considered to reach more aware choices, which can, in turn, lead to an individual's all-encompassing well-being.

### Information and overfitting

A crucial point in the decision-making process is how a single individual approaches the incoming information. We could notice how decisions become faster and faster and are made in a constantly changing environment. The amount of data grows exponentially, but despite its abundance it could be inaccurate, incomplete, or confusing. The consequent increasing spread of misinformation is more serious in some sectors with respect to others: a paradigmatic case is the increasing interest in the field of *Infodemiology*, the study of the determinants and distribution of health information and misinformation (Eysenbach, 2002). This phenomenon represents the importance in exploring the correlation between data, reasoning propensity, and decisions. When we think about thinking it is easy to assume that “more is better”; we may assume that having more data about the decision context can lead to a more accurate prediction of the future consequences of our choices, and thus to a better result. However, this cannot be always true. The question of how hard to think and how many factors to consider is at the heart of a thorny problem that statisticians and machine-learning researchers call *Overfitting* (Christian and Griffiths, 2016). If we were to have extensive, completely mistake-free data drawn from a perfectly representative sample, and a precise definition of exactly what needed to be evaluated, then the best approach would be using the most complex model available. In this ideal situation the only problem should be the presence of correlations among the different pieces of information used for building the predictive model. It is a well-known problem in statistics where it is defined as the *collinearity of the regressors* (Dormann et al., 2013). In the paradigmatic case of a dependent variable *Y* to be predicted by a set of independent variables *Xs* (regressors), the existence of mutual correlations between *Xs* creates a fundamental indeterminacy of the resulting model (Krishnan et al., 2007), causing unpredictable errors in subsequent applications. In any case, we can imagine that in an ideal case these problems can be faced by a preliminary factorization of the data set into mutually independent components (Xie and Kalivas, 1997), or by any other technique of prioritizing variable orders (Alhamzawi and Ali, 2018). Nevertheless, in a real situation that is far from being ideal, if we try to fit a given model to the actual data, a certain risk of incurring overfitting exists. In other words, overfitting poses a danger any time we are dealing with mismeasurement or environments that are vague, ambiguous, and ill-defined, as is commonly the case in a working setting and in everyday life due to the complex surroundings we live in. It is true that including more factors in a model will always, by definition, produce a better fit for our data. However, a better fit for the available data does not necessarily imply improved ability to generalize and thus better predict future cases that, by definition, are not available at the time the predictive model itself is constructed (Diaconis and Mazur, 2003; Christian and Griffiths, 2016).

Consequently, because any decision has to do with some kind of prediction of future outcomes, a better fit on actual data does not necessarily lead to a better decision. A model that is too simple can fail to capture essential aspects of the phenomenon studied; on the other hand, a model too complicated can become oversensitive to the particular adopted sample. Since the model is finely tuned to a specific data set, the resulting oversensitivity ends up intermingling a mixture of both general and idiosyncratic information relative to the specific sample, generating highly variable and consequently poor solutions. In this respect, it is worth noting that while the usual practice of splitting the whole data set into a “training” (from where the model is built) and a “test” set can be very wise, it only partially solves the overfitting problem. Successful models in science stem from the clear division of information into “sloppy” and “stiff” parts (in the jargon of data analysis). By focusing on solid information (usually consisting of very few control parameters) and leaving out the rest, it is possible to predict the behavior of very complex systems with good, and sometimes excellent, approximation (Transtrum et al., 2015; Giuliani, 2018; Ho et al., 2020). The inclusion of “sloppy” details marginally improves the adaptation of the model to the experimental data on which it is built, but at the expense of its generalization capacity.

It is possible to see the analogy with individual reasoning: an individual who excessively relies on an analytical approach, collecting as much data as possible and analyzing all details for their decision, will spend lots of energy in this effort and not necessarily select the best possible action. Being too analytical could lead to focusing specifically on details and losing the ability to consider the general purpose of the problem.

In other words, it is indeed true that knowledge originates from both deduction (move from the general to the specific), and induction (move from the specific, eventually many different specifics, to the general), but also strongly relies on *abduction* (Figueiredo, 2021). Abduction is the process of reasoning that goes from “the particular” to the general, but “the particular” is not a huge collection of data prepared to be analyzed by statistics, algorithms, and models. In abduction, “this particular” is a small set of specific and significant data that anchors our reasoning. It is the process used by doctors when they diagnose (Bird, 2010), by detectives when they try to resolve a criminal case, and by experts when they work. A paradigmatic example of the power of abductive reasoning in contrast to fully quantitative methods grounded in machine-learning is presented by Beaulieu-Jones, who shows the evidence of clinically driven decisions, based on heuristics approaches emerging from the personal expertise of the clinicians (Beaulieu-Jones et al., 2021).

From our point of view, the decision-maker who relies too much on the actual data can incur the dilemma of deciding based only on a specific sample of a much wider “population”, losing generalization power and possibly leading to a worse prediction and thus worse decisions, or even to inaction (Diaconis and Mazur, 2003). We can imagine a kind of threshold beyond which the logical and analytical approach of collecting and analyzing data becomes counterproductive. This happens because we stop considering properties “common” to a certain class of problems and start to model the singularities of the particular reference set that have no equivalent outside the narrow scope from which the data derives (Transtrum et al., 2015).

Being aware of this phenomenon could change our approach to the decision in some way: mitigation of the previously described drawback could derive from the adoption of a behavior that not only relies on analytical and logical reasoning built on collected information, but also considers other subjective components. Although these aspects are difficult to express, extract or demonstrate with objective data, they could, in some way, be formalizable which is one of the novelties introduced by this work.

### Foundational elements for a mathematical model of awareness

The development of a mathematical model of awareness can be addressed by starting from different perspectives and considerations (Friston, 2010). In this study we focused on philosophical, logical, cognitive and behavioral aspects; we aim to formally identify information flows and learning mechanisms that can allow individuals to initiate a process of increasing awareness instead of focusing on measuring conscious states and processes at the neural level (Modica and Rustichini, 1994; Heifetz et al., 2013; Karni and Vierø, 2017; Halpern and Piermont, 2020).

The first interesting contribution to the definition of a model of awareness can be found in philosophy and logic. According to Modica and Rustichini, a subject is *certain* of something when they know if it is true or false and *uncertain* when they know not its truth-value, and the subject's awareness of this not knowing is “conscious” uncertainty. On the other hand, a subject is *unaware* of something when they know not its truth value and is incognizant of their not knowing—they cannot perceive the object of knowledge, they are unable to mentally grasp it (Modica and Rustichini, 1999). The authors define *awareness* as the opposite of unawareness. Awareness includes both certainty and uncertainty, claiming that the concept of unawareness is a source of “ignorance” and distinct from uncertainty. They then propose the axiom of symmetry requirement for awareness: an individual can be aware of a proposition φ if and only if they are aware of its negation (*not-*φ). In other words, φ and ¬φ (*not-*φ) are either perceived together or not at all. A logical definition of awareness is given in terms of knowledge: assuming *A*φ means “the individual is aware of the propositional φ” and *K*φ “the individual knows φ”, the logical formulation of awareness is:


$$\begin{array}{l} A\varphi =\;K\varphi \vee \left(\neg K\varphi \wedge K\left(\neg K\varphi \right)\right)\;\forall \varphi \; \end{array}$$

which is PL-equivalent to:


$$\begin{array}{l} A\varphi =\;K\varphi \vee K\left(\neg K\varphi \right)\;\forall \varphi \; \end{array}$$

where PL stands for propositional logic. A subject who is aware of fewer things than another is not at all necessarily less capable of logical reasoning, those are two separate concepts. Nevertheless, it is possible the individual may not be capable of making some deductions precisely due to their unawareness: for example, if they are unaware of *q* they will not be able to deduce the concept of “*p implies q*” from knowledge of *p*, which would otherwise be doable to one who is aware of *q*. Therefore, awareness can improve people's decisions.

These aspects are relevant when choosing the right model class to characterize the processes that lead to increasing awareness—in our research that being the Markov Decision Process which shall be described in the model section. Notably, it assumes that increased awareness is the result of personal effort and clear focus with this specific objective in mind. Further, becoming aware reduces ignorance even if it does not reduce uncertainty, something intrinsically considered in the stochasticity of the model.

Awareness is a term that is often interchangeably used in different contexts, however, the literature dealing with awareness can be organized around three core concepts (Carden et al., 2022). The first is *cognitive awareness* (Papaleontiou-Louca, 2003) which identifies awareness as an accurate and deep understanding of an individual's perception, thinking, and actions. The second perspective argues that awareness exists on the multiple levels of consciousness and unconsciousness. Here, we consider awareness as an end-stage experience that results from the filtering and processing of several possible experiences happening simultaneously in our bodies and brains (Vaneechoutte, 2000). The third definition considers awareness with regard to recognizing others' feelings (Beck et al., 2004) and taking into account one's impact on others.

Further, whilst dealing with an individual's self-awareness, which is a relevant component of the developed model, two typologies are identified in the literature: “intra” and “inter” personal self-awareness (Carden et al., 2022). These are linked to an individual's own internal state and their impact on others, respectively, which can be further broken down into seven different components.

(1) *Beliefs and Values*. Beliefs Refer to the Conviction or Acceptance That Some Propositions About the World Are True, Especially Without any Proof, Whereas Values Denote the Hierarchical, Dynamic, and Abstract Attribution of a Degree of Importance to Something, Reflecting One's Judgment About What Is Important in Life. (2) *Internal Mental State* That, in Turn, Includes the sub-Components of *Feelings and Emotions* and *Thoughts and Cognitions* (Scherer, 2005; Wessinger and Clapham, 2009). (3) *Physical Sensations* Corresponding to Physiological Responses Manifested as Reactions in the Body. (4) *Personality Traits*, Reflecting the Individual's Stable and Consistent Characteristic Patterns of Thoughts, Feeling, and Behaviors. (5) *Motivations and Desires*, Which Are Related to Personal Drivers and Mental Directions. (6) *Behavior* Corresponds to an Action That Others see or Hear Individuals Displaying, Thus It Is an Interpersonal Component. (7) *Other Perception* Corresponds to how an Individual Is Perceived by Others, and the Ability to “Receive” Feedback. In Conclusion Self-Awareness can be Defined as:

*Self-awareness consists of a range of components, which can be developed through focus, evaluation, and feedback, and provides an individual with an awareness of their internal state (emotions, cognitions, physiological responses) that drives their behaviors (beliefs, values, and motivations) and an awareness of how this impacts and influences others*.

In developing a mathematical model, these findings allow self-awareness as a fundamental factor of awareness to be built in, including emotions, cognitive processes, motivations, believes, evaluations and feedback. All these elements can easily be embedded into a Markov Decision Process.

All the factors outlined above have a heavy impact on many other aspects of our social life. The ability to use tacit knowledge and intuition is common and necessary anytime people need to make decisions in complex environments that are future-oriented, highly uncertain, difficult to forecast and lacking in information (Mintzberg, 2000). For example, in the field of leadership and management, rational decision-making skills are required to enable processing available information clearly and logically and thus permitting accurate perception and interpretation of the incoming events, which can sometimes lead to creative and innovative solutions (Prietula and Simon, 1989). Nevertheless, apart from this type of knowledge is essential to consider that managers routinely make decisions based on knowledge grounded in experience and could use intuitive decision strategies, especially under high-stress conditions (Sayegh et al., 2004). Tacit knowledge—the work-related practical know-how acquired through direct experience and instrumental in achieving goals important to the holder (Brockmann and Anthony, 2002)—is not easily recognized or acknowledged, but it can be a key factor in enhancing the quality of strategic decisions.

The sensible application of tacit knowledge can partially fill information gaps ameliorating the efficiency of the decision process (Brockmann and Anthony, 1998). Further, self-awareness is now seen as a critical component in leadership and career success, joining the skills of team interaction development, effective coordination and collaboration (Dierdorff and Rubin, 2015), and non-conflictual and sensible leadership (Axelrod, 2005).

## Developing a mathematical model of awareness

In this section we introduce a mathematical model that can incorporate the main factors summarized in the previous sections. According to the statements discussed in Section Decision-making processes: an overview, we have applied the class of models referred to as Markov Decision Processes, which have been deemed suitable to describe human decision-making under uncertainty (Rangel et al., 2008), including autopiloted decisions (Landry et al., 2021) and addictive behavior (Mocenni et al., 2019).

### Sequential decision models and Markov Decision Processes

Each day people make many decisions that have both immediate and long-term consequences. Decisions must not be made in isolation, today's decisions impact tomorrow's and tomorrow's the day after; if one does not account for the relationship between present and future decisions and present and future outcomes, one may not achieve overall good performance. The *Sequential Decision Models* (SDMs) consider both outcomes of current decisions and future decision-making opportunities under some kind of uncertainty (Puterman, 2014).

**Figure 2:**
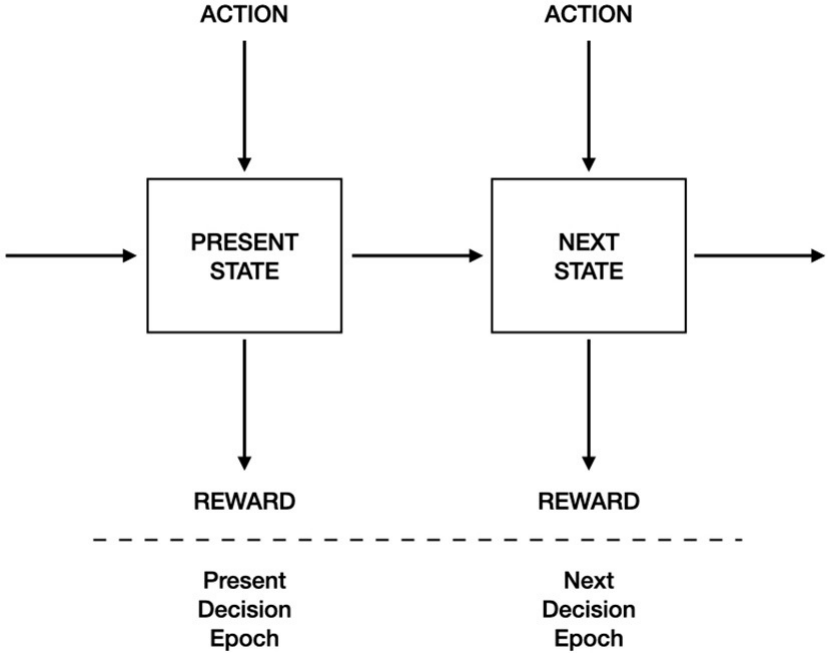
Schematic representation of a Sequential Decision Model.

In a sequential decision-making model at a specified point in time (that is also called “decision epoch”) the *Decision-maker* (DM) observes the current state of the system, and based on this state, chooses an action among the ones available in that state. The choice produces two results: the DM receives an immediate reward (or incurs a cost) and the system evolves to a possibly new state in the next decision epoch. At this subsequent point in time, the DM faces a similar problem as schematized in Figure 2. The key ingredients of this sequential decision model are:

- A set of decision epochs;
- A set of states;
- A set of available actions (which can be different in different states);
- A reward (or cost) function depending on state and action;
- A set of state transition probabilities depending on state and action.

We usually assume that the DM knows all these elements at the time of each decision, thus they constitute the amount of explicit information available.

At each decision epoch, the DM performs a choice, and they can have a *policy* that provides the most favorable action in each possible state; the implementation of a policy generates a sequence of rewards (or costs). The sequential decision problem consists in finding a policy before the first decision epoch, which maximizes a function of the rewards' sequence (or minimizes the costs' sequence). A policy that accomplishes this task is defined as optimal and relative to the specific individual and function considered.

*Markov Decision Processes* (MDPs) are a particular class of SDMs in which actions, rewards, and transition probabilities solely depend on the current state, and not on occupied states and actions chosen in the past. In other words, the current state incorporates the entirety of the DM's past: it is the result of all their previous decisions, related outcomes, and experience gained from them. In this work, we try to characterize some dynamics of the awareness-raising process. Therefore, we assume awareness is a dynamic process (characterized by a sequence of states with a certain dynamic in time) involving the DM's experiences, filtered by his perspective, beliefs, values, actual state, and choices, through different reasoning attitudes. Moreover, in an MDP, we simultaneously have the presence of a decision-maker's choice and uncertainty regarding its outcomes, as always happens with our decisions due to uncontrollable factors.

This model should be a good trade-off between realism and simplicity: broad enough to account for realistic sequential decision-making problems while simple enough to allow it to be understood and applied by different kinds of practitioners.

It is worth stressing that MDP, even if incorporates in a given state *s*_*t*_ the DM's entire past, represents a future evolution uniquely dependent on the current state and is completely independent of the particular trajectory that reaches state *s*_*t*_ at time t. Only *a posteriori* reflection by a DM on the trajectory of past experience builds (in the long run) both awareness and tacit knowledge. Here we recognize the presence of two dynamics with very different time scales. The short timescale of MDP, ending up in the terminal decision, and the long timescale (we can think of the physical analogy of a capacitor) that generates both awareness and tacit knowledge by reflecting on past stories of successes and failures that can, in turn, be of use for future decision processes.

### Model formulation

#### Time and state

As mentioned above, the adopted model belongs to the framework of Markov Decision Processes (MDPs) and considers a discrete and finite time horizon of length *T* in which, to each *time-epoch, t*, corresponds to a moment of making a relevant decision—which needs some kind of reasoning process and is not purely automatic and routine. Since the life of an individual is limited, it is reasonable to consider a finite time horizon. The *state, s*_*t*_ ∈ (0,1), of the individual is a representation of their level of awareness at each time-epoch *t* and belongs to the set *S* represented by the discrete closed interval (0,1) with a step size of 0.01. In this way, a unique definition of awareness is not explicitly given, which would be a very difficult task, it is simply said that awareness is a state of the individual which determines their well-being from a global point of view and has a considerable impact on their choices. This paper is mainly focused on discussing how awareness can be accounted for by a mathematical model more than giving an explicit definition of it. It introduces an attempt to model some mechanisms underlying the process of aware decision-making rather than quantifying the effective awareness of individuals in some way, which could be a herculean task. By considering an MDP, the current state incorporates the DM's complete history so that their awareness is a state, in some way, embodying all of the individual's past: from their personality, values, beliefs developed over their lifetime, to their education and past experiences.

#### Reasoning propensity

The *reasoning propensity, p*_*r*_ ∈ (0,1) embeds the specific attitude in processing the information about the problem, and represents the trade-off between the two reasoning modalities: *analytical* and *intuitive*. This combination depends upon different individual factors like age, character, beliefs, values, desires, education, experience, and so on; it varies from individual to individual but can also change in the same individual throughout their lifetime. The reasoning propensity takes values in a continuum between the two extreme attitudes (Allinson and Hayes, 1996), called *intuitive* (*p*_*r*_ = 0) and *analytical* (*p*_*r*_ = 1), assuming in this way that both are always involved, to different degrees, in any decision. These two modalities refer to the dichotomy between rationality and intuition, as Section Decision-making processes: an overview brings to light.

#### Policy and decision

The reasoning propensity affects the *policy*, μ of the individual. Generally speaking, a policy is a function that prescribes the action to make for each possible state at any time instant, and is represented by a matrix of dimensions [|S| x T]. It can be somewhat complicated to shape different situations. Therefore, the policy turns out in the *decision, u*_*t*_, which belongs to the open interval *U* = (0,1), so that the more analytical the choice, the higher the value of *u*_*t*_. We have that:


$$\begin{array}{l} u_{t}\;=\;\mu \left(s_{t},t\right)\;\forall \;s_{t}\in S\;and\;t\;=\;1,\ldots ,\;T \end{array}$$

The choice leads to two results: the DM receives a reward, and the system possibly evolves to a new state.

#### State evolution and transition probability functions

The DM's state, s_t_, evolves according to:


$$\begin{array}{l} s_{t+1}\;=\;f\left(s_{t},u_{t},w_{t}\right) \end{array}$$

That is, the future level of awareness of the individual depends on the current state, the choices they mak e, and its outcome, which is subject to some uncertainty represented by *w*_*t*_, the *stochastic variable* related to a state transition. We assume, for simplicity, that the state can remain the same or increase and decrease by only one step, in this way *w*_*t*_ belongs to the set *W* = {1, 0, −1}, indicating, respectively, the possibility that the state increases, remains constant or decreases by making a decision *u*_*t*_. The presence of uncertainty affecting the outcomes of the decision due to uncontrollable elements in the environment makes the state evolution and the rewards sequence stochastic. There exists a known *transition probability*, function of *u*_*t*_, specified in a matrix P of dimensions [|U| x 3]. In particular, *P* has the form:


$$\begin{array}{l} P\;=\;\left[P_{1}\left(u_{t}\right)\;P_{0}\left(u_{t}\right)\;P_{-1}\left(u_{t}\right)\right] \end{array}$$

Each one of the three columns of *P* specifies the probability that the state increases, remains constant, and decreases, respectively. In other words:

- *w*_*t*_ = 1 *with probability P*_1_(*u*_*t*_) → *Forward probability*
- *w*_*t*_ = 0 *with probability P*_0_(*u*_*t*_) → *Stationary probability*
- *w*_*t*_ = −1 *with probability P*_−1_(*u*_*t*_) → *Backward probability*

In this way, the system dynamics can be re-written as:


$$\begin{array}{l} s_{t+1}\;=\;s_{t}+\;w_{t} \end{array}$$

Notice that all the elements in the matrix *P* are values representing a probability, and are subject to two conditions:


$$\begin{array}{l} {{0\leq P}_{w}\left(u_{t}\right)\leq 1\;\forall \;w\in W\;and\;P}_{1}\left(u_{t}\right)\\ \;+P_{0}\left(u_{t}\right)+P_{-1}\left(u_{t}\right)\;=\;1\;\forall \;u_{t}\in U \end{array}$$

The *stationary probability P*_0_ has been defined as a constant value, notably all simulations consider:


$$\begin{array}{l} P_{0}\left(u_{t}\right)\;=\;0.1\;\forall \;u_{t}\in U. \end{array}$$

It incorporates the DM's resistance to change their level of awareness, and depending on their characteristics, can be considered “inertia”. The *forward probability* results from the linear combination of two fixed functions exploiting the cases of intuitive and analytical reasoning.

**Figure 3:**
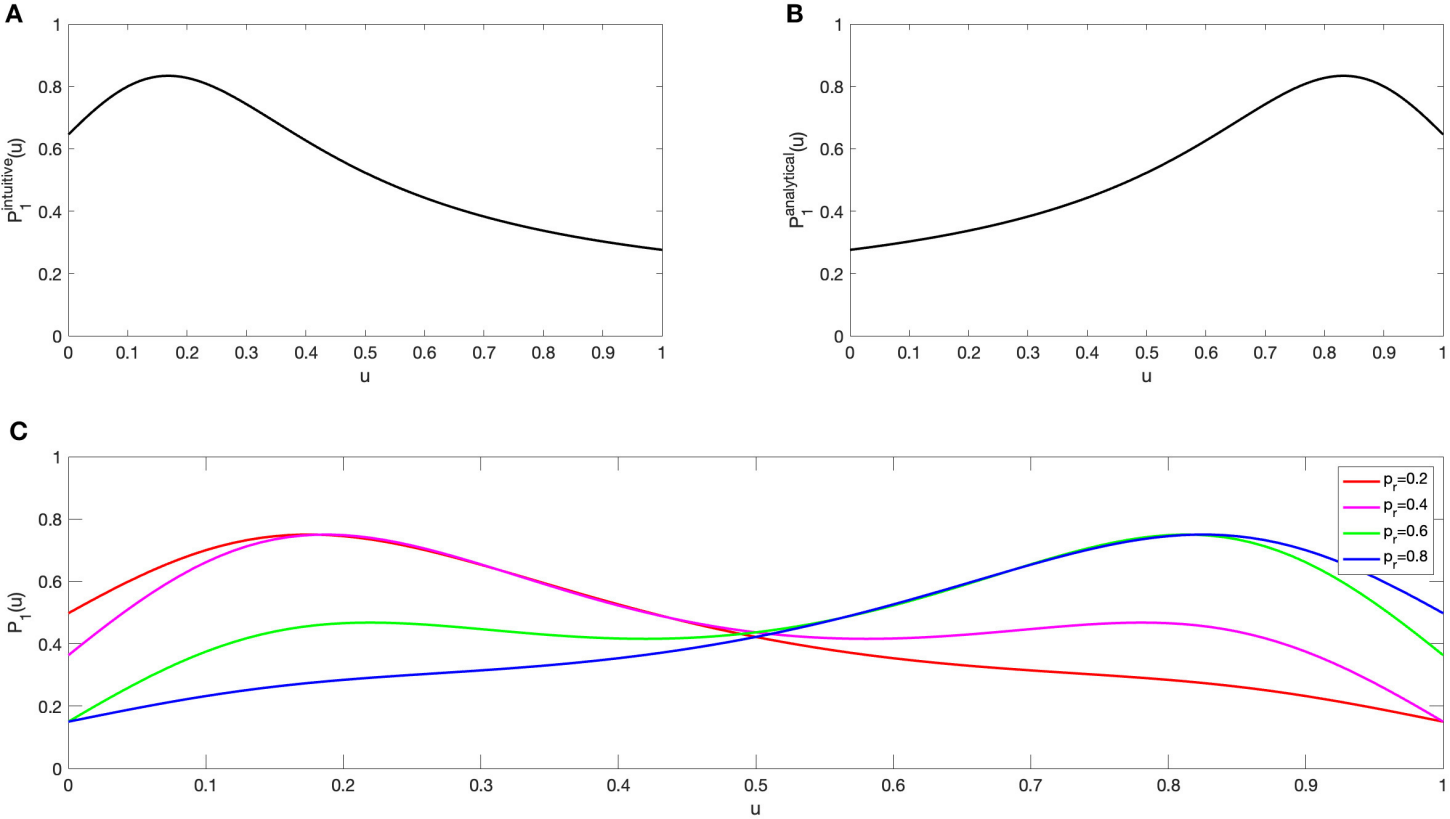
Forward transition probability. **(A, B)** indicate the forward transition probabilities of an intuitive and analytical individual, respectively. These two functions are linearly combined using the specific individual's reasoning propensity *p*_*r*_. Some examples of forward transition probability functions for different *p*_*r*_ are shown in **(C)**.

Figure 3 shows the functions considered for the forward probability in the—only theoretical—cases of a complete analytical (Figure 3A) and a complete intuitive (Figure 3B) individual. The first one starts with a low value and then increases as the decision becomes more analytical. It reaches the maximum when the reasoning is highly analytical, and then, for bigger values of *u*_*t*_, the probability starts to decrease. This is a representation of the *overfitting* phenomenon described in Section Decision-making processes: an overview, exploiting the fact that an excessively analytical approach to reasoning could also turn out to be counterproductive. The second function has an opposite behavior: the more intuitive the reasoning (the smaller *u*_*t*_), the bigger the probability of increasing the state. This is because the individual thinks to have access to personal abilities, not related to cognition, allowing their level of awareness to increase by using an appropriate degree of intuition; this has to do with the personal confidence in tacit knowledge. It is possible to consider that a minimum level of analyticity is indispensable to understand the framework of the decision in this case; otherwise, intuition loses contact with the reality of the decisional problem, becoming only a fantasy. An excessively intuitive individual may act without considering the context from which the decisions come, and the decision could be ineffective. We must note that all these transition functions reflect the DM's different points of view; we are putting ourselves in the shoes of the individual. It is very difficult to consider a transition probability function that generically specifies the probability of increasing the state of individual awareness without depending on any such kind of assumption.

The two basic functions (Figures 3A,B) have been designed so as to represent the different theoretical assumptions exposed in Section Decision-making processes: an overview, including the drawbacks of being excessively analytical or intuitive. Certainly, different functions can be considered as long as they are capable to incorporate the same phenomena.

As we mentioned above, any real DM mixes, to some extent, these two modalities according to a personal proportion represented by their reasoning propensity *p*_*r*_. The effective forward transition probability of the DM, i.e., the probability of increasing the state's level, is computed as the convex combination of the two fixed functions—forward transition probabilities in the only theoretical case of complete analyticity or intuitiveness of the individual—using the reasoning propensity *p*_*r*_ as coefficient:


$$\begin{array}{l} P_{1}\left(u_{t}\right)\;=\;p_{r}P_{1}^{analytical}\left(u_{t}\right)+\left(1-p_{r}\right)P_{1}^{intuitive}\left(u_{t}\right) \end{array}$$

Figure 3C shows the influence of different values of *p*_*r*_ on the transition probability *P*, as described in the legend.

Finally, the *backward probability* is defined starting from the first two as:


$$\begin{array}{l} P_{-1}\left(u_{t}\right)\;=\;1-\left(P_{1}\left(u_{t}\right)+P_{0}\left(u_{t}\right)\right) \end{array}$$

#### Rewards

The problem now arises of how to define a function that grants the individual a reward by selecting choice (*u*_*t*_) instead of another and being in a certain state *s*_*t*_. Is it important to maintain the focus on what are we trying to explain with the model; that is: investigate the dynamic underlying the process of awareness-raising. In fact, as exposed in Section Decision-making processes: an overview, the dynamic of awareness-raising emerges from personal effort and motivation. Moreover, as human agents, we are accustomed to operating with rewards that are so sparse we only experience them once or twice in a lifetime, if at all. Most goals of modern life—a good job, a house, a family, a happy life—are so abstract, complex, and far into the future that they do not provide useful reinforcement signals. Despite this, people continuously make choices in their lives, applying what psychologists call *intrinsic motivation* or *curiosity*. Motivation/curiosity have been used to explain the need to explore the environment and discover novel states. Similar to what also happens in reinforcement learning, intrinsic motivation/rewards become critical whenever extrinsic rewards are sparse (Pathak et al., 2017). In our case, intrinsic motivation refers to reaching higher states of individual awareness, which can be linked to reaching sparse, temporary, distant and extrinsic life goals.

Mathematically the reward function consists of two parts: a *stage reward* explicitly depending on state and choice, and implicitly on the stochastic variable w_t_. The second is a fixed *terminal reward, r*(*s*_*T*_), which the DM incurs at the last time-epoch T. The stage reward linearly depends on the current state and the choice, with constant and positive coefficients α and β:


$$\begin{array}{l} r\left(s_{t},u_{t}\right)\;=\;\alpha s_{t}-\beta u_{t} \end{array}$$

It is reasonable to assume that the individual's current level of awareness has a positive influence on an individual's whole life, so living with a higher level of awareness can improve well-being on all levels (physical, psychological, emotional, and so on). Consequently, the equation incorporates a positive dependence on the current level of awareness so that the higher it is, the better the individual's life is overall. On the other hand, rational/analytical reasoning is resource-consuming because it requires the acquisition of some kind of data about the problem and the possible alternative solutions, and requires time to analyze and elaborate all the data. Intuitive reasoning, as also revealed in Pascal's thought, is *effortless* and not resource- consuming. Therefore, the more the decision implies analytical reasoning the more resources it needs in terms of time, personal energy, and monetary resources. This translates into a negative dependence of the reward on the choice *u*_*t*_, because the higher *u*_*t*_ is the more analytical the reasoning of the DM, and so the more resources consumed. Although the current version of the model assumes a linear form for the step-reward function, to explain the thoughts behind its formulation in a simple way other typologies functions are possible and may be more suitable.

**Figure 4:**
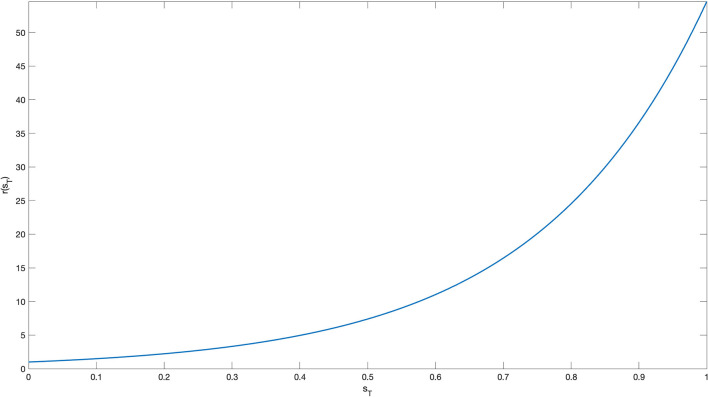
Terminal reward as a function of the level of awareness at the final time instant.

For the same reasons set out in the previous point, we can assume that the terminal reward the DM incurs at time T increases with the value of the ending state (Figure 4). It has been considered an exponential function that “tries to push” the final state as high as possible, providing a considerably different reward between ending in a “high” state rather than in a “low” state.

Here we can also notice how tacit knowledge can be thought of as deriving from the sedimentation of past cases into an “experience capacitor”. The terminal rewards derived from past cases lose their specific reference to the actual situation from which they stemmed, and contribute to the creation of a “good practices” repository no longer linked to a particular situation but a “broad spectrum of cases”.

#### Future weights

Equal rewards at different time-instants have a different value for the individual. Therefore, factor δ weigh ing future rewards has been introduced. Different applications and aspects referred to this consideration are studied in detail in Section A model extension: Including individual emotions.

#### Backward penalty

In the end, we considered a vector γ of dimensions [3 x 1] to give possible different weights to the cases of increasing, maintaining constant, or decreasing the state, respectively. We assume different values of γ in different simulations, accounting for different attitudes of the DM—for example strongly penalizing the eventuality of decreasing awareness.

### Habitual decisions

Once the general structure of the model and the meaning of its parameters are defined, it is possible to consider how the resulting model can be applied to different situations shaped through different policies. In the following sections we present two ways to find suitable policies aimed at solving the decision problem. The first, proposed in this paragraph, refers to the most basic and simplest mechanism governing an individual's *habitual decisions* (or *choices*), the ones made with little to no effort and without conscious control (Landry et al., 2021). They consequently assume that the DM does not have any self-awareness, so that decisions spring only from their habits as automatic, non-conscious mechanisms.

As mentioned in the previous section, the DM has their own reasoning propensity p_r_ indicating how much the decision-making process is intuitive or analytical. It is possible to assume that this is the only characteristic governing habitual decisions, considering a naïve policy, that is accordingly called *habitual* or *usual policy*, defined as:


$$\begin{array}{l} \mu \left(s_{t},t\right)\;=\;e_{t}\;\forall s_{t}\in S\;and\;t\;=\;1,\ldots ,T \end{array}$$

Where *e*_*t*_ is normally distributed with mean *p*_*r*_ and standard deviation σ fixed to a constant value, for example 0.3, represents the fact that any individual's decision always encompasses the processing of information regarding a problem in a similar way, more or less analytically. However, a certain variation in the choices has been considered around the value representing the propensity of reasoning, supplied by other uncontrollable contextual factors which are the real drivers of the decision, making the individual unaware of being able to effectively decide the value to choose. These factors represent a source of uncertainty, influencing the choice, and can drive it far from the effective *p*_*r*_, highlighting the case in which people are not synchronized with their effective reasoning propensity.

Ultimately, this policy's structure shapes the case of the DM's unaware decisions. It is possible to see some conceptual similarities to the UMDPs (Unaware Markov Decision Processes) which represent an attempt to introduce the concept of unawareness in the framework of Markov processes (Halpern et al., 2010). A common idea is the restriction of the set of possible actions, even if implemented in different ways, reflecting the unawareness of the DM regarding an entire set of possible actions. In the policy described above, beyond this kind of unawareness, the individual is also unaware of their effective reasoning propensity, assuming that the effective choice randomly selects a value around it. In this way, the unaware choices are not completely random but reflect a kind of coherence of the individual and, on the other hand, shape an unawareness of what is the real *p*_*r*_.

In this work, this structure is mainly applied as a term of comparison to evaluate the effect of introducing an individual's self-awareness on the choices.

### Self-aware decisions

**Figure 5:**
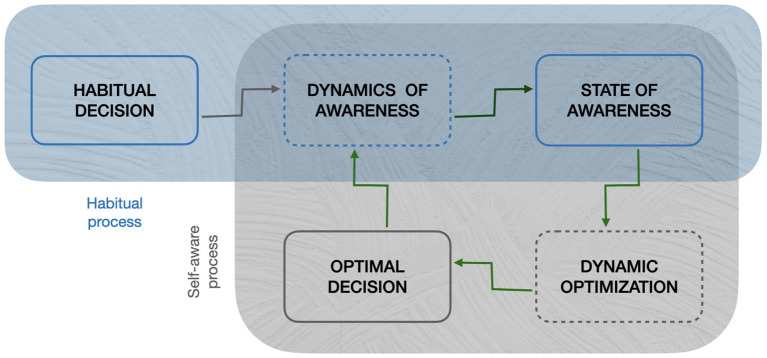
A schematic logical representation of the model. The blue (green) circle indicates the structure in the case of *habitual* (*self-aware* or equivalently *optimal*) decisions and their intersection. The dashed borders of the two blocks relative to the dynamics symbolize the uncertain factors impacting the dynamic in time.

The second structure that is proposed represents a first attempt to incorporate the concept of self-awareness in the process. If we think about self-awareness, we could imagine that it is an element deriving from some kind of self-observation—a “third person” perspective from a metacognitive point of view (Drigas and Mitsea, 2020)—and that has a consequent impact on the action/decision. Mathematically it can be represented by a *feedback loop*, according to the logical representation of Figure 5.

Accordingly, a component that modifies the policy has been introduced by additionally observing the form of the transition probability function, current state, and time epoch. In this way, the DM is allowed to modify their usual, automatic process by shifting from their habitual to a new policy that mathematically results from a maximization process. This introduces the possibility of mitigating the habitual tendencies of the individual by modifying the policy.

This feedback is mathematically embedded in an optimization process, intended to maximize the sequence of rewards. Due to the linear dependence of the reward on the level of awareness, it is also thus modeling the fact that self-awareness results from a personal effort.

#### Computation of the optimal policy

As previously mentioned, a policy is *optimal* when it maximizes a certain objective function which, in this case, is the cumulative sum of the rewards incurred at each time epoch. One of the methods that can be applied to compute the optimal policy in an MDP is the *Stochastic Dynamic Programming* (SDP) algorithm, choosing the action which maximizes the sum of the current reward and the expected future rewards at each stage. Mathematically we can say that the following problem must be solved:


$$\begin{array}{l} \;\underset{\mu }{\max}\;E\left[\overset{T-1}{\underset{t\;=\;0}{\sum }}r\left(s_{t},\mu \left(s_{t},t\right),w_{t}\right)\;+\;r\left(s_{T}\right)\right]\\ s.t.\;s_{t+1}\;=\;f\left(s_{t},\mu \left(s_{t},t\right),w_{t}\right)\;t\;=\;1,\ldots ,T \end{array}$$

Considering a finite time horizon of length *T*, a decision is *not* made at time *T*, so that the DM's last choice is at time *T*-1, and the final time instant is used to fix a terminal reward the DM incurs at time *T, r(s*_*T*_*)*. From there it is possible to recursively reconstruct the optimal policy by exploiting Bellman's *Principle of Optimality* (Bellman and Drayfus, 2015) which affirms that “*an optimal policy has the property that whatever the initial state and initial decision are, the remaining decisions must constitute an optimal policy with regard to the state resulting for the first decision”*. The original problem can be decomposed into a recursive series of easier sub-problems, considering a shorter time horizon from τ to *T* and a given initial state :


$$\begin{array}{l} V_{\tau }\left(\underline{\text{s}}\right)\;=\;{\text{max}}_{\mu_{\tau }\ldots \mu_{T-1}}\left[\overset{T-1}{\underset{t\;=\;\tau }{\sum }}r\left(s_{t},\mu \left(s_{t},t\right),w_{t}\right)\;+\;r\left(s_{T}\right)\right]\\ \;s.t.\;s_{t+1}\;=\;f\left(s_{t},\mu \left(s_{t},t\right),w_{t}\right)\;s_{\tau }\;=\underline{\text{s}}\;,\;t\;=\;\tau ,\ldots ,T \end{array}$$

where $V_{\tau }\left(\underline{\text{s}}\right)$ is the value function that indicates the optimal reward cumulatively obtained considering the sub-problem with time horizon τ*, …, T* and initial state. Starting with τ = *T*-1 and then decreasing the value of one unit each time, it is possible to recursively calculate the optimal policy for which the current optimal value can be seen as the sum of the expected stage reward and the expected optimal value function at the next time instant:


$$\begin{array}{l} V_{\tau }\left(\underline{\text{s}}\right)\;=\;r\left(\underline{\text{s}},\;\mu \left(\underline{\text{s}},\tau \right)\right)+V_{\tau +1}\left(f\left(\underline{\text{s}},\mu \left(\underline{\text{s}},\tau \right),w_{t}\right)\right) \end{array}$$

This expression in our case can be expanded considering that *w*_*t*_ can assume three values with probabilities specified in matrix *P*. So, it becomes:


$$\begin{array}{l} V_{\tau }\left(\underline{\text{s}}\right)\;=\;r\left(\underline{\text{s}},\;\mu \left(\underline{\text{s}},\tau \right)\right)+\delta [\gamma_{1}V\left(\underline{\text{s}}+1\right)P\left(\mu \left(\underline{\text{s}},\tau \right),1\right)\\ +\gamma_{2}V\left(\underline{\text{s}}\right)P\left(\mu \left(\underline{\text{s}},\tau \right),2\right)+\gamma_{3}V\left(\underline{\text{s}}-1\right)P\left(\mu \left(\underline{\text{s}},\tau \right),3\right)], \end{array}$$

where 0 < δ < 1 is the weight given to the next-instant reward, and γ = [ γ_1_, γ_2_, γ_3_] is the vector of the different weights given to the possibility of increasing, remaining constant, and decreasing the next state, respectively. Each constant coefficient γ_*i*_ belongs to (0,1).

It is worth remembering that the transition probabilities explained in matrix *P* depend on the reasoning propensity *p*_*r*_ of the individual, and this determines the effective shape of the curve representing forward, stationary, and backward probabilities as a function of the decision (*u*_*t*_) suggested by the policy.

### A model extension: Including individual emotions

Immediate emotions (also called *visceral factors* in economics) play a critical role in the intertemporal choice modifying the utility of an action, leading people to behave in ways that appear to greatly discount the future, ways that individuals themselves can sometimes see as contrary to their own self-interest (Loewenstein, 2000).

In this description, we can identify three basic aspects that could help model emotions: their relationship with time, with perception (utility) or a reward, and the fact that they could also be counterproductive. In the proposed model the ideal place to insert emotions seems to be in factor δ that weights future rewards. It permits connecting immediate emotions to the perception of the future and the value of the rewards.

We especially claim that emotions do not necessarily hurt an individual's choice but can also “help” them. We can equate emotions to the role played by “temperature” in simulated annealing optimization models (Bertsimas and Tsitsiklis, 1993); in order to escape eventual local minima during the optimization process the simulated annealing algorithms allow for a certain degree of stochasticity that could inhibit the system to take the “most convenient move” during the optimization process. This mirrors the role of temperature that can make the system exit from a potential hole, the temperature (i.e. the degree of stochasticity) decreases during the process and goes to a minimum near the end of the process so as to not destroy the reach of the optimal solution. It is worth noting that, at odds with simulated annealing, our model does not incorporate an explicit decreasing trend of temperature (contribution of emotion) but an equivalent effect is reached by introducing a dependence on awareness's dynamic along the process that in turn can make the emotions somewhat less relevant.

The entity of the role played by emotions depends on the level of awareness of the individual. At a low level of awareness, emotions prevail on individual reasoning, so one is completely driven by choices in search of instant gratification (independent of the reach of the actual target). In this condition, the future will have very little weight on one's choice, which can be detrimental because people behave in a way that is contrary to their self-interest. Contrarily, this dynamic is not present when the individual reaches a high level of awareness in which one could consider emotions freely and peacefully, and could, in some way enhance a benefit from the choice.

Another aspect to considered in the computation of δ is the relationship between future weight of choice and the age of the individual, in such a way that the older the individual, the bigger the weight they give to future rewards. An older individual with less time to live consequently gives more importance to each possible moment in the future; in contrast, a younger individual could weigh the present with less consideration of the future.

Mathematically, this additional extension changes the equation defined in the paragraph regarding the computation of the optimal policy, which becomes:


$$\begin{array}{l} V_{\tau }\left(\underline{\text{s}}\right)\;=\;r\left(\underline{\text{s}},\;\mu \left(,\tau \right)\right)+\delta \left(\underline{\text{s}}\right)\frac{t}{T}[\gamma_{1}V\left(\underline{\text{s}}+1\right)P\left(\mu \left(\underline{\text{s}},\tau \right),1\right)\\ \;+\gamma_{2}V\left(\underline{\text{s}}\right)P\left(\mu \left(\underline{\text{s}},\tau \right),2\right)+\gamma_{3}V\left(\underline{\text{s}}-1\right)P\left(\mu \left(\underline{\text{s}},\tau \right),3\right)], \end{array}$$

with time horizon τ*, …, T* and an initial (known) state $\underline{\text{s}}$.

The term δ(*s*) is introduced in the model to insert an emotional component. It indicates a modification of the structure of δ which, until now, was a constant value but is now considered as a function of the state (see Figure 7A). Moreover, it reports a linear dependence on time *t*, where the term $\frac{1}{T}$ is a scale factor. Summarizing, the new term embeds the impact of emotions in the decisions as a factor which enforces the expected value of the future reward when either the awareness level increases, or the time horizon reaches its maximum, or both.

## Numerical results

The next step was to carry out numerical simulations to apply the different structures corresponding to the habitual and self-aware policies outlined in the previous section in order to evaluate the evolution of the dynamic. To do this, it must be also specified:

- The *number of simulations, N*, to perform. Each of them with a time horizon of length *T*.
- An *initial state s*_0_ for each simulation. It can be fixed to a particular value to evaluate the dynamic starting with a specific level of the state or can be computed as a random value extracted from a discrete, uniform distribution that takes values from 0 to 1.
- Notice that *t*_0_ and *s*_0_ are related to the instant when the observation starts, they are not intended as absolute values yet always have a relative connotation.
- For each time instant in each simulation we need a *realization* of the random transition variable *w*_*t*_ which takes values in *W* = {1, 0, −1} according to the probability functions in *P* evaluated at *u*_*t*_; in fact, the DM implements, at each time, a choice according to the policy μ they choose (*u*_*t*_ = μ(*s*_*t*_,*t*)).

Performing *N* = 3,000 simulations and analyzing the average value in all of them, we can see what happens to the trends of state and rewards.

**Figure 6:**
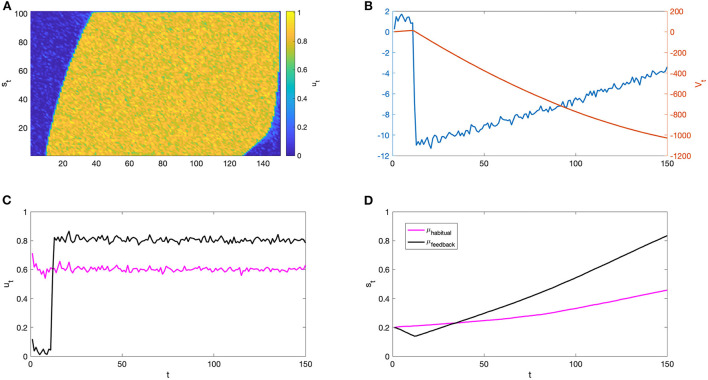
Some results of the simulations. We considered an individual with *p*_*r*_ = 0.6, low noise on the policy (σ = 0.08), and a low initial state (*s*_0_ = 0.2). The other model's parameters were fixed to α = 1, β = 1.5, γ = [3 1 0.1], and δ = 0.75. **(A)** reports the matrix of the optimal policy computed in the presence of the feedback loop: it indicates the decision *u*_*t*_ (indicated by the color) to perform for each combination of time epoch and state. **(B)** reports the step (blue) and cumulative (red) rewards in the presence of feedback. **(C, D)** highlight the different evolution of the states and decisions considering habitual (magenta) and self-aware (black) behavior, respectively.

Some numerical results are reported in Figure 6, obtained by considering an individual with *p*_*r*_ = 0.6. The habitual policy for such a kind of individual is constantly centered around 0.6 with, in this case, low noise. It means that the choice of the individual always has roughly 60 percent analyticity of reasoning. From the optimal policy's matrix (Figure 6A), one can see that the optimal policy suggests starting with a low level of *u*_*t*_ and then increase it to 0.8 (Figure 6C). The lack of observation of transition probabilities and the level of state creates, in the habitual case, a decreased rise in state, which at ending time reaches 0.5, whereas with the introduction of feedback the state is able to saturate near the maximum state (Figure 6D). We have chosen this particular case to discuss in light of the phenomenon that the state initially decreases in the presence of feedback. It is generally possible to notice that the state with the feedback loop monotonously increases and is higher with respect to the other. These results are explained in our first publication on that topic (Bizzarri and Mocenni, 2022), where the embryonic idea of comparing habitual and optimal strategies in human decision-making has been presented, while the mathematical model, including the concept of overfitting, tacit knowledge and emotion, have been introduced in the present paper for the first time. However, by considering a different parameter setup we can notice that even if the state temporarily decreases, the presence of a feedback loop allows for a change in the trend, reaching values that are even higher than in the case of habitual behavior. The step reward has a similar trend with an initial decrease and then a more rapid increase than in the habitual case (Figure 6B—blue line).

### Including emotions in the model

It is possible to evaluate the impact of embedding emotions of the individual by performing some simulations and correspondingly modifying the computation of the optimal policy in the presence of a feedback loop, as exposed in Section 4.5.

**Figure 7:**
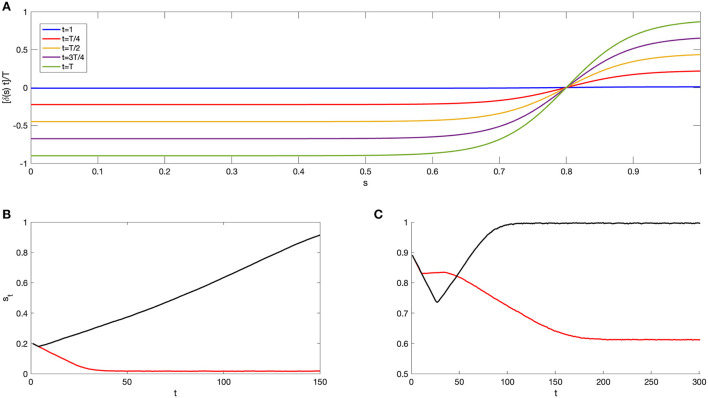
Analytical individual with emotions. **(A)** reports the new term $\delta \left(s\right)\frac{t}{T}$ defined as a function of the state *s*_*t*_ for different specific values of time: *t* = {*t*_0_, *T/4, T/2, 3T/4, T}*. **(B, C)** analyze the effect of emotions on an individual with *p*_*r*_ = 0.8, starting with a low (s_0_ = 0.2) and a high (*s*_0_ = 0.9) initial state, respectively. The red line indicates the behavior in presence of emotion whereas the black line in absence of emotion (considering a constant δ = 0.75). In both cases, the feedback loop is included, modeling self-aware decisions. The other model parameters are fixed to α = 10, β = 20, γ = (3.7, 1, 0.01), σ = 0.08.

It is possible to observe that a highly analytical individual starting from a low state manifests a decrease of the state in the presence of an emotional factor, as described in Figure 7B. This is due to the new form of δ*(s*_*t*_*)* (Figure 7A), which has a negative value for a low state of awareness, claiming that in this case the presence of emotions greatly effects future discounting which could also turn out as harmful (in case of negative values of δ). On the contrary, after gaining a certain level of awareness, δ starts to increase reaching a value near 1, meaning that an individual with a high level of awareness does not make any distinction between the present and future.

Figure 7C demonstrates the possibility that emotions could enhance the state evolution: in this case, emotions are helpful in increasing the state evolution over transient times, until it stabilizes at a constant value (*s*_*t*_ ~ 0.7). This behavior appears when considering a longer time horizon, where *T* is set at 300.

**Figure 8:**
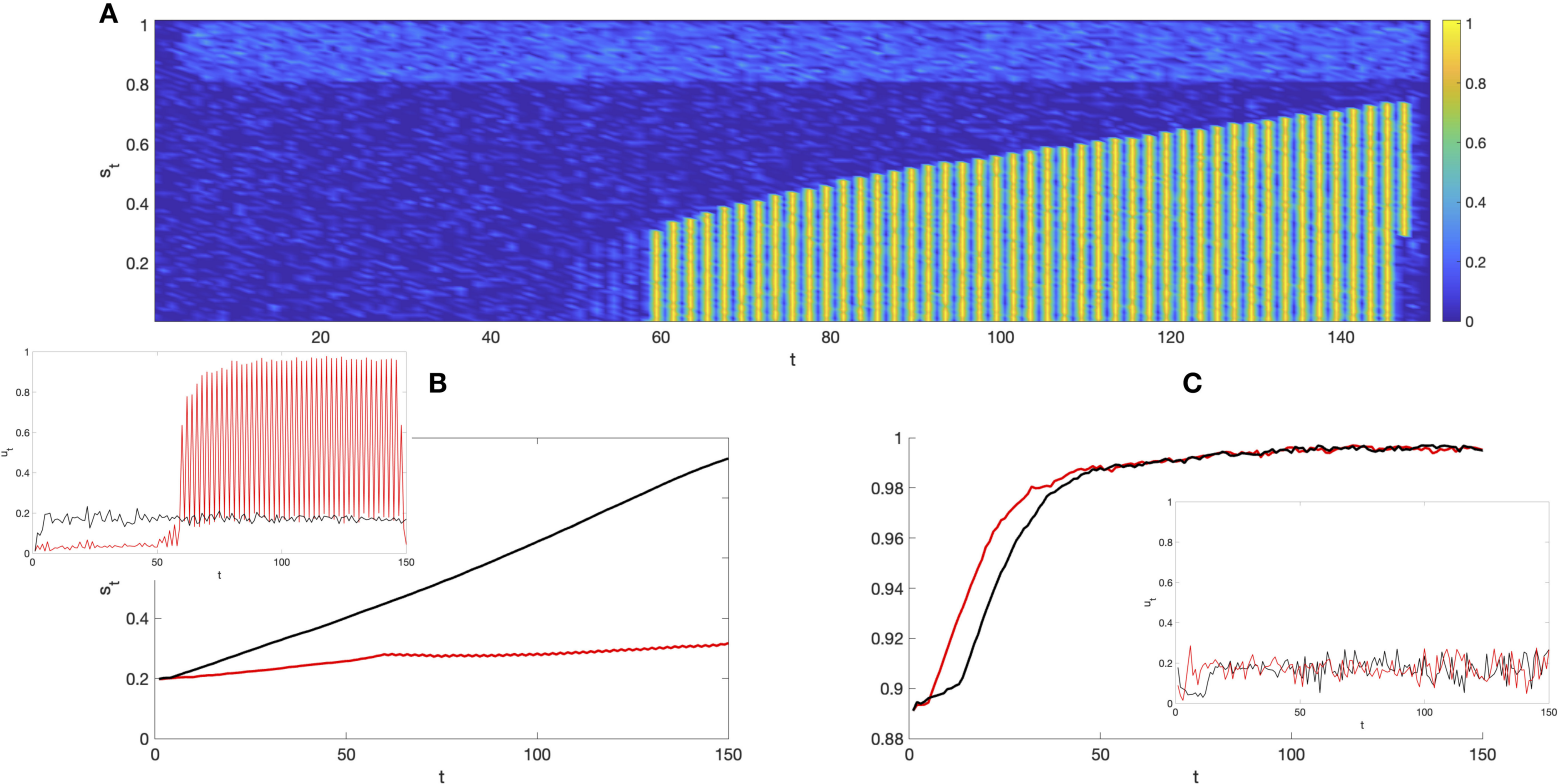
Intuitive individual with emotions. Now we shall consider an individual with *p*_*r*_ = 0.2, analyzing, as before, the trends due to emotion starting from a low initial state. **(A, B)** analyze the effect of emotions starting low (*s*_0_ = 0.2). **(A)** reports the optimal policy whereas **(B)** demonstrates the state evolution and, in the insert, the effective choice over time. **(C)** considers a high (*s*_0_ = 0.9) initial state and shows the state dynamic and, in the insert, the efficient choice. The red line indicates behavior in the presence of emotion whereas the black line in absence of emotion (considering a constant δ = 0.75). In both cases the feedback loop is included, modeling self-aware decisions. The other model parameters are the same used in Figure 7.

The helpful effect of emotions starting from a high *s*_0_ is more evident in the following when considering an intuitive individual (Figure 8C).

In the case of a highly intuitive individual, the additional emotive aspect creates an oscillatory behavior when considered at a lower initial state (Figures 8A,B). This oscillation makes it impossible to choose a stable value of *u*_*t*_, which oscillates between high and low values. Consequently, it stops the growth of the state.

As also highlighted in the analytical case, the presence of emotions at high states enhances the evolution of the state that increases faster than without emotions (see Panel C, where the red line is over the black one).

## Discussion

The aim of this research was to investigate how to integrate facets like tacit knowledge, intuition, emotions, awareness, and self-awareness into a mathematical model of decision-making, going beyond the classical analytical perspective. These factors have been considered within the framework of a richer conception of the decision-maker, and their multi-faceted effects are intensely analyzed. Even though all of them have been studied and described from a theoretical point of view in different fields of investigation, a modeling formalization is still missing, and this is the novelty that the present work introduces: the possibility to incorporate all these different aspects into a model of decision-making. A still very primitive framework has been proposed allowing the integration of non-analytical factors into a coherent frame. We achieve such integration by taking into account qualitative definitions of non-analytical factors that stem from different fields of investigations, and quantifying them within the framework of a generalized Markov Model decision process. In this context, the importance of a modeling approach resides in its capacity to focus on the principal and essential factors involved in a process, in this case of decision-making, concisely and practically describing each of them and meanwhile maintaining an overview on the entire phenomenon. We hope that this initial step could lead to further exploration, and deepen each aspect to increase the model details, such as interaction with others, which some preliminary results have been already founded by the authors.

This study does not have a specific psychological connotation, instead it attempts to integrate current cognitive psychology research with the more varied—and inherently uncertain—outcomes of human decision-making and could contribute to the introduction of new aspects, expanding research in this field, such as self-observation and the ensuing emotion, and the use of unexplicit information in the decision. The psychological (and philosophical) dimensions of awareness were, in turn, deeply investigated by Drigas and Mitsea (2020) in terms of metacognition by stressing the need for a reflexive act in which the decision maker acts as a “third person”, retrospectively evaluating their previous strategies and consequently building up a “tacit knowledge reservoir”. It is not without consequence that the authors insert one of the basic pillars of metacognition, “the internalized knowledge that awakens and drives humans towards independence and the fulfillment of each one's potential” (Drigas and Mitsea, 2020).

There is a large consensus about the presence of two distinct mechanisms in order to tackle the relationship between information and the decision process that we have called intuitive and analytical; which here have been developed, suitably revisited and extended. First, the presence of the phenomenon of overfitting derives from excessive confidence in the analytical approach, which leads the individual to focus on the details of a specific sample that is part of a much wider “population”, losing generalization power and potentially moving towards poor predictions and thus poor decisions. We could imagine a kind of threshold beyond which the logical and analytical approach of collecting and analyzing data becomes disadvantageous. Indeed, beginning to model the singularities of the particular reference set that have no equivalent outside the narrow scope from which the data may prevent considering properties “common” to a certain class of problems. We mathematically formulate this phenomenon by introducing the *forward probability transition* of an analytical individual (see Figure 3B), claiming that after a certain threshold of u_t_, a further level of analyticity results as a decrease in the probability of reaching a higher value of awareness. On the other hand, the *forward probability transition* of an intuitive individual claims that the more intuitive the reasoning (the smaller u_t_), the bigger the probability of increasing the state is until a given lower bound is reached. This happens because the individual thinks they have access to personal abilities, distinct from cognition, allowing the level of awareness to increase by using a kind of unexplicit acquaintance related to tacit knowledge. Thus, the idea expressed by Pascal's *esprit de finesse*, an effortless ability available to each individual but often unknown, is accounted for by our model. Tacit knowledge is inherently difficult to express, extract or demonstrate with objective data but, despite these setbacks, it could possibly be formalized which is one of the novelties introduced in this work.

The model questions a purely analytical “one-size-fits-all” approach, stressing the importance of considering the uniqueness of each single individual who could in any case autonomously change their habits thanks to the implementation of a kind of self-observation mechanism, and recognizing the effectiveness of their personal and unique repository of tacit knowledge.

Moreover, the specificity of an MDP allows bidirectional vision with a look to the future in the evaluation of the optimal choice at each time instant, and a retrospective reconstruction of the entire sequence of choices and the dynamics of the state enabling different perspectives of observation. Considering time an independent variable it is possible to observe the mechanisms by which the state evolves. The model does not provide a univocal definition of awareness, but rather considers it as the result of a series of processes, as described above, which can allow the individual to retrospectively observe the process that led them to be the person they are today.

In the end, interesting aspects arise from the introduction of emotions in the model. We have started from the consideration that emotions impact an individual's intertemporal choice, modifying perceived utility and leading people to behave in ways that seem to disregard the future, thus sometimes damaging the individual themselves. All these aspects are considered in the definition of weight δ that the DM attributes to future rewards. Typically, when included, emotions are evaluated as “noise” to avoid or minimize. The different point of view proposed in this work claims that emotions do not necessarily hurt an individual's choice, they can be “helpful”. This depends on one's level of awareness, which can be considered as strictly related to the ability to manage and integrate emotions in decision-making, and in turn enhance the individual's awareness. From the simulations it is possible to appreciate the validity of the above considerations. Starting from a low initial level of awareness in both analytical and intuitive individuals (Figures 7B, 8B), emotions have a damaging effect. The difference is that in the first case the state irrevocably decreases to minimal one, whereas in the second it stopped at a local value without increasing anymore. The analytical case can be interpreted as the typical idea that emotions disturb choice, but, in our model this is only true when considering low states of awareness. At low states the analytical individual is not able to relate with and manage emotions, and this reflects their state decreasing to zero. In the intuitive case, on the other hand, the state stops increasing due to the appearance of an oscillatory dynamic in choice, where the decisions oscillate from a low to a high value without maintaining a constant trend in time. Emotions create an unstable dynamic that does not permit constant and long-lasting decisions over time, resulting in a stationary state. The interesting aspect is that at high levels of awareness, these behaviors do not manifest, and indeed emotions can exert a beneficial influence (Figures 7B, 8C). This is more evident in the intuitive case, whereas in the analytical case they improve in the transient before the state stabilizes to a constant value. This is an indication that emotions are not necessarily a nuisance in the decision process.

Another relevant result has to do with the mathematical formalization of emotions that resonates with the concept of awareness, typical of oriental traditions as connected to the capacity of living in the “*present moment*”, where the individual is focused on the present occurrence of experience without interference from past or anticipated images (Kang and Whittingham, 2010). It is not by chance that the exhortation of “living in the present moment” is shared by diverse philosophies, from monastic Christian (Merton, 2010) to mindfulness techniques (Carpenter et al., 2019). Living in the present moment does not mean to be prey to the search of immediate gratification (that means weighing the future with a negative delta), rather it corresponds to the capacity to give an equal weight to each instant (in our case having a delta that reaches one). This is exactly what happens in our model by increasing time and *s*_*t*_, thus becoming older and more aware.

## Conclusions

This work incorporates essential drivers for human decisions, analyzing their reciprocal relations and influences into a model grounded in the Markov process. From the analytical/intuitive dichotomy to the inclusion of tacit knowledge and the impact of emotions, all the different facets of a decision have been discussed from both a theoretical and a mathematical point of view. Individual awareness emerges from the comparison between habitual strategies and the ones sprung from the addition of an individual self-awareness feedback, and its dynamic nature can be appreciated from an individual's retrospective observation. Moreover, the impact of emotions is re-thought with an explicit dependence on the level of awareness of the individual, so that their conception that emotion is a noise to be filtered is mitigated by the consideration that it is true at low state of awareness, and can thus be enhancing for aware individuals. From an epistemological point of view, our aim was to demonstrate how commonly first sight decisions are taken for granted (resonating in diffuse expressions like “clinical eye”), and cannot be considered as a purely “emotional” process opposing “strictly analytical strategies”; instead, they are the result of a long and largely tacit learning process. This concept was already present in the words of Blaise Pascal nearly 400 years ago but progressively forgotten by specialist literature. Here, we give a proof-of-concept of the possibility to insert this kind of knowledge into a mathematical model alongside the philosophical issues we think this result could help solve, from problems encountered by machine intelligence to facing problems relevant for biomedical applications (Gavrishchaka et al., 2019; Beaulieu-Jones et al., 2021).

The limitations are the obvious and inherent ones in creating a mathematical model of such complex phenomena as human decisions and awareness are. Mathematically, modeling awareness is a herculean task, and the model will inevitably be “sloppy” due to the inability to enclose the immensity of human thought into a few functions that are, at best, a stimulus for a more realistic consideration of decision-making process.

One way to overcome the above limitations could be by testing the model in reality, for example developing surveys and designing experiments that can allow for the collection of estimations of the model's parameters and adapt the model to specific cases. A second crucial step forward in model- understanding is to also consider the presence of interactions among individuals. Taking a cue from some preliminary results obtained by the authors in this direction, there is a plan to investigate the effects on the decision process and awareness evolution introduced by interaction within a network of individuals and the different impacts due to the structure of the relationships.

## Data availability statement

The original contributions presented in the study are included in the article, further inquiries can be directed to the corresponding author.

## Author contributions

CM, AG, and FB: Conceptualization and writing. CM and FB: Mathematical modeling, simulation, and software development. All authors listed have made a substantial, direct, and intellectual contribution to the work and approved it for publication.
